# 12 Hip involvement in juvenile idiopathic arthritis: frequency and associated factors

**DOI:** 10.1093/rheumatology/keac496.008

**Published:** 2022-10-07

**Authors:** Houssem Tbini, Safa Rahmouni, Soumaya Boussaid, Ahlem Ben Ammou, Samia Jemmali, Sonia Rekik, Khaoula Zouaoui, Hela Sahli, Mohammed Elleuch

**Affiliations:** 1 Department of Rheumatology, La Rabta Hospital, Tunis, Tunisia; 2 University of Tunis El Manar

## Abstract

**Background:**

Juvenile idiopathic arthritis (JIA) is a pediatric rheumatic disease with several subgroups. The hip is frequently affected. The frequency of this involvement can reach 50% especially in the severe and destructive forms. Arthroplasty may be indicated in advanced cases.

**Objectives:**

To assess the frequency and associated factors with hip involvement in (JIA).

**Methods:**

We conducted a retrospective study including adults with long-standing JIA according to the International League of Associations for Rheumatology (ILAR) criteria over a period of 28 years (1994–2022). Demographic, clinical, biological, and radiographic data were collected. These parameters were compared according to the presence or absence of hip involvement.

**Results:**

A total of 29 Patients were enrolled (12 men and 17 women), the mean age was 35.69 ± 11.72 [18–61] years. The mean age of disease onset was 11.10 ± 4.25 [2–16] years. The average diagnostic delay was 52.96 ± 95.97 [0–336] months. The average disease duration was 24.48 ± 12.76 [1–47] years.

Sixteen patients had a polyarticular form. Mean CRP values were 42.74 ± 63.37 [2–218] mg/l, a biological inflammatory syndrome was present in 19 cases. Rheumatoid factor, ACPA and anti-nuclear antibodies were observed in 12, 7 and 5 cases respectively. At least one extra-articular manifestation was noted in 16 cases.

Hip involvement was noted in 14 patients (48.3%). It was bilateral in 64.3% of cases (*n* = 9). Twenty-three hips were affected in total (56.9%). Hip involvement was diagnosed 14.5 ± 9.37 [1–28] years after disease onset. Arthroplasty was performed on 10 hips with a delay of 201.60 ± 104.75 [108–348] months between diagnosis of JIA and surgery.

Hip involvement was associated with male gender in our study (75% *vs* 29.4%; *p* = 0.016). On the other hand, our study showed that age, age at onset, diagnostic delay, symptoms duration, smoking, BMI, extra-articular manifestations, CRP, rheumatoid factor, antinuclear antibodies, ACPA and erosive character were not associated with hip involvement.

**Conclusion:**

Our study showed that hip involvement is common in JIA, mainly in male patients. It usually occurs late in the disease course. Since hip involvement is a cause of disability and functional impairment, it should be assessed regularly.

